# The effects of probiotics supplementation on *Helicobacter pylori* standard treatment: an umbrella review of systematic reviews with meta-analyses

**DOI:** 10.1038/s41598-024-59399-4

**Published:** 2024-05-02

**Authors:** Zihan Yang, Yueyang Zhou, Ziying Han, Kun He, Yuelun Zhang, Dong Wu, Hongda Chen

**Affiliations:** 1 State Key Laboratory of Complex Severe and Rare Diseases, Department of Gastroenterology, Peking Union Medical College Hospital, Chinese Academy of Medical Sciences and Peking Union Medical College, Beijing, 100730 China; 2 Peking Union Medical College Hospital, Medical Research Center, Chinese Academy of Medical Sciences and Peking Union Medical College, Beijing, 100730 China; 3 Clinical Epidemiology Unit, Peking Union Medical College Hospital, Peking Union Medical College, Chinese Academy of Medical Sciences, Beijing, 100730 China

**Keywords:** Probiotics, *H. pylori*, Eradication, Side effects, Antibiotics, Microbiology, Gastroenterology

## Abstract

*Helicobacter pylori* infection, a worldwide health issue, is typically treated with standard antibiotic therapies. However, these treatments often face resistance and non-compliance due to side effects. In this umbrella review, we aimed to comprehensively assess the impact of probiotics supplementation in different preparations on *Helicobacter pylori* standard treatment. We searched PubMed, Embase and Cochrane Central Register of Controlled Trials in the Cochrane Library from inception to June 1, 2023, to identify systematic reviews with meta-analyses that focused on eradication rates, total side effects and other outcomes of interest. The most comprehensive meta-analysis was selected for data extraction. AMSTAR 2 was used to assess quality of meta-analyses. Overall, 28 unique meta-analyses based on 534 RCTs were included. The results suggests that probiotics supplementation with pooled probiotic strains was significantly associated with improved eradication rates (RR 1.10, 95% CI 1.06–1.14) and reduced risk of total side effects (RR 0.54, 95% CI 0.42–0.70) compared with standard therapy alone. Single-strained or multi-strained preparation of probiotics supplementation showed similar results. Despite *Bifidobacterium spp.* showing the highest potential for eradication, the study quality was critically low for most meta-analyses, necessitating further high-quality research to explore the optimal probiotic strains or their combinations for *Helicobacter pylori* treatment.aq_start?>Kindly check and confirm the edit made in article title.

## Introduction

*Helicobacter pylori* (*H. pylori*), a Gram-negative and transmissible bacterium, infects the epithelial lining of the stomach in approximately half of the global population, which equates to an astonishing estimate of roughly 4.4 billion individuals, consequently imposing a significant medical and social burden^1^. The infection predominantly triggers a lifelong, chronically progressive gastric inflammation, potentially leading to a variety of diseases such as peptic ulcer, gastric atrophy, gastric intestinal metaplasia and, in more severe instances, gastric cancer or mucosa-associated lymphoid tissue lymphoma^2^. Following international consensus established since 2017, it is advised that *H. pylori* be eradicated promptly upon diagnosis^3^. Currently, the most widely recommended standard therapy involves either triple or quadruple therapeutic regimens given for 7–14 days, including a proton-pump inhibitor (PPI) and two distinct antibiotics such as clarithromycin combined with either amoxicillin or metronidazole, optionally complemented by bismuth salts^3,4^. However, the past two decades have witnessed a concerning global increase in antibiotic resistance of *H. pylori*, which has coincided with a continuous decline in the success rates of eradication therapies^5,6^. Moreover, common side effects of antibiotics such as diarrhea, nausea, and vomiting contribute to patient non-compliance, thereby resulting in less satisfactory eradication rates when using standard therapy^7^.

Probiotics, defined as live microorganisms, serve a vital function in health maintenance, particularly within the digestive system, when consumed in adequate amounts^8^. Numerous benefits that probiotics confer have been unveiled by researchers, including immunomodulation, protection against pathogens, and improved barrier function^9^. Given these properties, probiotics have garnered significant interest for their potential application in the management of *H. pylori* infections. Commonly used probiotics in medical treatments encompass genera like *Lactobacillus*, *Bifidobacterium*, *Saccharomyces*, along with various combined formulations. Evidence have found that probiotics could inhibit *H. pylori* colonization from both in vivo and in vitro studies^10,11^. Several meta-analyses have further investigated the potential of probiotics to enhance *H. pylori* eradication rates and reduce side effects when used in conjunction with standard treatment^12–14^. However, these findings have not gone without skepticism^15^, impeding the integration of probiotics into the routine clinical management of *H. pylori* treatment. Furthermore, the effectiveness of various probiotic strains, or their combined preparation, may vary within standard *H. pylori* treatment. It is crucial to understand that the efficacy of probiotics is cardinally strain-specific^16^, not genus-specific, which underscores the necessity for precise identification and utilization of effective strains in clinical applications.

Therefore, in this article, to systematically assess the impact of probiotic supplementation on *H. pylori* treatment, an umbrella review was conducted by scrutinizing meta-analyses limited to randomized controlled trials (RCTs). We aimed to tackle the state of the art in this scientific arena, providing a comprehensive and up-to-date perspective.

## Methods

This umbrella review was conducted in accordance with the Preferred Reporting Items for Overviews of Reviews (PRIOR) statement^17^. The study protocol was prospectively registered on PROSPERO (CRD42023444917) (https://www.crd.york.ac.uk/PROSPERO/).

### Search strategy

The search strategy was crafted by a medical information specialist (H.C.) in collaboration with the review team. The literature search was conducted independently by two researchers (Z.Y. and Y.Z.) in the following electronic databases from inception to June 1, 2023, with the keywords including “helicobacter pylori”, “probiotics” for all relevant systematic reviews with meta-analyses: PubMed, Embase, Cochrane Central Register of Controlled Trials in the Cochrane Library. The search words and/or Medical Subject Heading (MeSH) terms are listed in Table S1. Additional searches regarding this topic were conducted on Google Scholar and Web of Science. There was no language restriction. The titles and abstracts retrieved from the database underwent meticulous screened, with meta-analyses that met the inclusion criteria being identified by full text reading. In the event of any discrepancy in the literature selection process between the two reviewers, a third author (K.H.) intervened for resolution. Besides, the reference lists of all included articles were manually searched to identify any additional eligible studies that might otherwise have been overlooked.

### Eligibility criteria

We aimed to amalgamate data from systematic reviews and meta-analyses to provide a more expansive perspective on the impact of probiotic supplementation on *H. pylori* eradication therapy. Therefore, we selected *H. pylori* eradication rates, total side effects, specific gastrointestinal (GI) side effects, and other nonspecific side effects as outcomes of interest. Hereinto, specific GI side effects range from diarrhea, nausea, vomiting, nausea/vomiting, loss of appetite, constipation, abdominal distension, bloating, epigastric pain, abdominal pain, taste disturbance, metallic taste, to flatus, while other nonspecific side effects include skin rash, stomatitis, dizziness, palpitation, and blurred vision.

Studies fulfilling the following criteria were eligible in our umbrella review: (a) studies should be systematic reviews with meta-analyses of RCTs published in any language; (b) study populations should have undergone systematic and standardized eradication treatment including triple therapy, bismuth-containing quadruple therapy, or sequential therapy for *H. pylori i*nfection, irrespective of the number of times of infection and age; (c) patients in the control group should have received standard therapy with or without a placebo, while patients in the experimental group should have received standard therapy with probiotics; (d) availability of relative information on successful *H. pylori* eradication rates. Exclusion criteria were as follows: (a) irrelevant studies, duplicate publications, animal studies, systematic reviews without meta-analyses, comments, letters, case reports, conference abstracts, or editorials; (b) study populations were restricted to children.

When only a single meta-analysis investigated the outcome of interest, it was directly chosen for result presentation and discussion. However, when several meta-analyses probed the same outcome of interest, the most comprehensive study bearing the most complete outcome data was selected. This strategy, employed to prevent data duplication, reinforce the rigor of the assessment, and streamline the interpretation of results, has been used in other published umbrella reviews when overlapping meta-analyses exist^18–21^. Selection was primarily determined by the following key factors: (a) the methodological quality as gauged by the AMSTAR 2 assessment; (b) the date of publication; (c) the number of included RCT studies and the number of patients. Generally, when multiple meta-analyses investigated the same outcome of interest, we selected the one with the highest AMSTAR 2 assessment as a first priority for data extraction. If multiple meta-analyses with the identical AMSTAR 2 assessment investigating the same outcome of interest were published more than three years apart, we selected the most recent one for data extraction, which typically features the largest sample size. If multiple meta-analyses were conducted within the same three-year span, we prioritized the meta-analysis that incorporated the greatest number of RCT studies or patients. By using aforementioned filtering strategies, two reviewers (Z.Y. and Z.H.) independently selected the most comprehensive study for each outcome of interest. In all cases, rationale for selection was recorded. For conflicting evaluations, the third reviewer (H.C.) was consulted to solve the dispute and a final decision was made by the majority of the votes.

### Data extraction

Two reviewers (Z.Y. and Y.Z.) independently screened all records and resolved conflicts by consensus. Relevant data were extracted from included articles by one reviewer (Z.Y.) and peer reviewed by a second reviewer (Y.Z.). Extracted details included study characteristics (e.g., title, publication year, number of RCT studies, number of patients), estimated summary effects (odds ratio or risk ratio with 95% confidence intervals) in the intention-to-treat (ITT) analysis, study quality assessments and study limitations. Furthermore, the model of effect (random or fixed), heterogeneity (I^2^ statistic and Cochran’s Q test P value), and publication bias assessment (P value of Egger’s test or funnel plot) for each outcome of interest were extracted.

### Quality assessment

Two independent reviewers (Z.Y. and Y.Z.) conducted quality assessment of all included studies, and any discrepancies were solved via discussion to reach the consensus. First, the reviewers evaluated the methodological quality of the included meta-analyses by using The Measurement Tool to Assess systematic Reviews (AMSTAR) 2, a critical appraisal tool in assessing the quality of systematic reviews and meta-analyses^22^. The AMSTAR 2 assessment contains 16 items and generates an overall quality rating based on weaknesses in critical domains. The quality was determined to be “high,” “moderate,” “low,” or “critically low.” Additionally, the reviewers categorized the evidence of outcomes into five classes, guided by the evidence classification criteria: Class I (convincing evidence), Class II (highly suggestive evidence), Class III (suggestive evidence), Class IV (weak evidence), and NS (non-significant)^23^.

### Patient and public involvement

Patients and the public were not involved in the planning, design, and implementation of the study, as this study used secondary data. No patients were asked to advise on interpretation or writing up of the manuscript.

## Results

### Literature search results and study selection process

**Figure 1:**
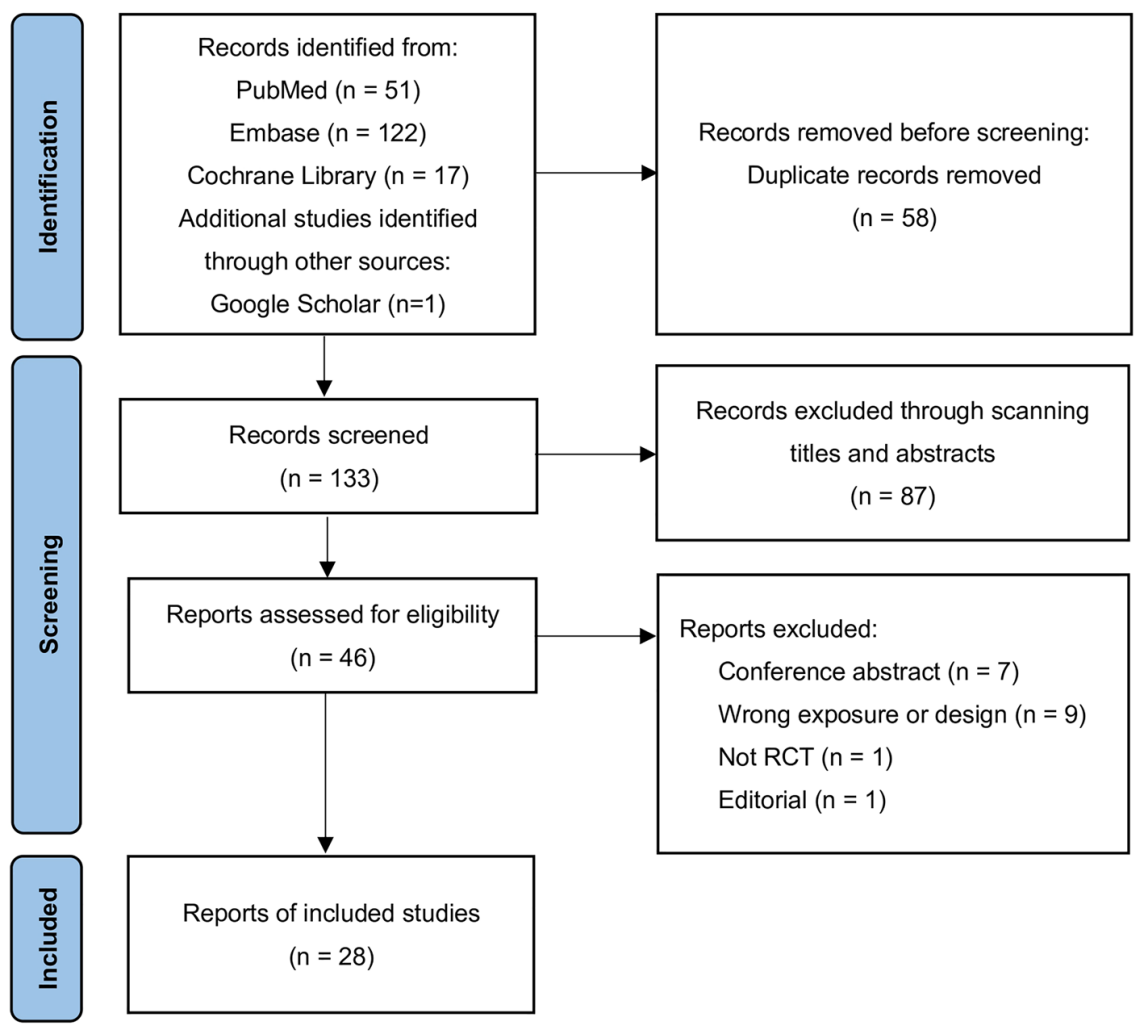
Preferred reporting items for systematic reviews and meta-analyses flow chart for study inclusion. The initial search retrieved 191 records (identification), among which 58 were removed as duplicates. After the screening of title and abstract, 133 records were chosen for full-article reading (screening). Finally, 28 meta-analyses met the inclusion criteria of the umbrella review.

Our systematic literature search yielded a total of 191 distinct articles. Subsequently, we discerned 28 systematic reviews with meta-analyses of RCTs that satisfied the eligibility criteria^12–15,24–47^. Details of the selection protocol are presented as a diagram in Fig. 1. Agreement between the two reviewers (Z.Y. and Y.Z.) for study selection was almost perfect (κ = 0.88, 95% confidence interval 0.67–1.08; P < 0.001).

Our preliminary review on the impact of probiotic supplementation alongside standard *H. pylori* treatment reveals that single-strained probiotics, particularly those containing *Lactobacillus spp.*, *Bifidobacterium spp.*, and *Saccharomyces spp*. are more commonly used. These probiotics have shown promise in increasing the success rate of *H. pylori* eradication and in mitigating side effects. While recent research points to varying levels of effectiveness among probiotics^47,48^, consensus is still lacking regarding the optimal probiotic choice for *H. pylori* eradication. Therefore, in this umbrella review, we examine the effectiveness of probiotics in different preparations that might lead to distinct outcomes of interest.

**Figure 2:**
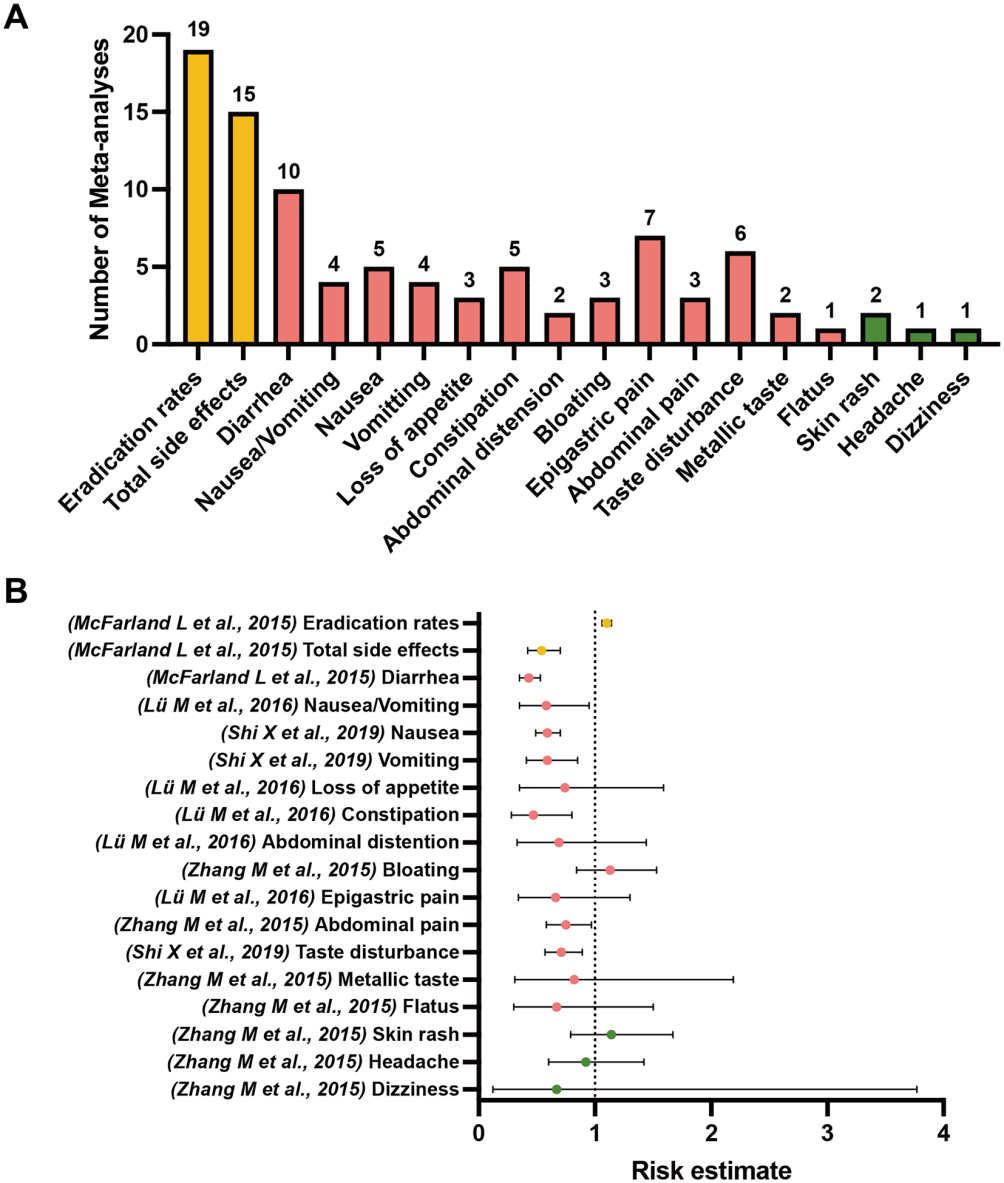
(**A**) Number of systematic reviews with meta-analyses included which investigated outcomes of interest between *H. pylori* standard treatment supplemented with pooled probiotic strains and the control. (**B**) Simplified forest plot summarizing evidence for the association of *H. pylori* standard treatment supplemented with pooled probiotic strains with outcomes of interest in systematic reviews with meta-analyses categorized as the most comprehensive for each outcome.

**Figure 3:**
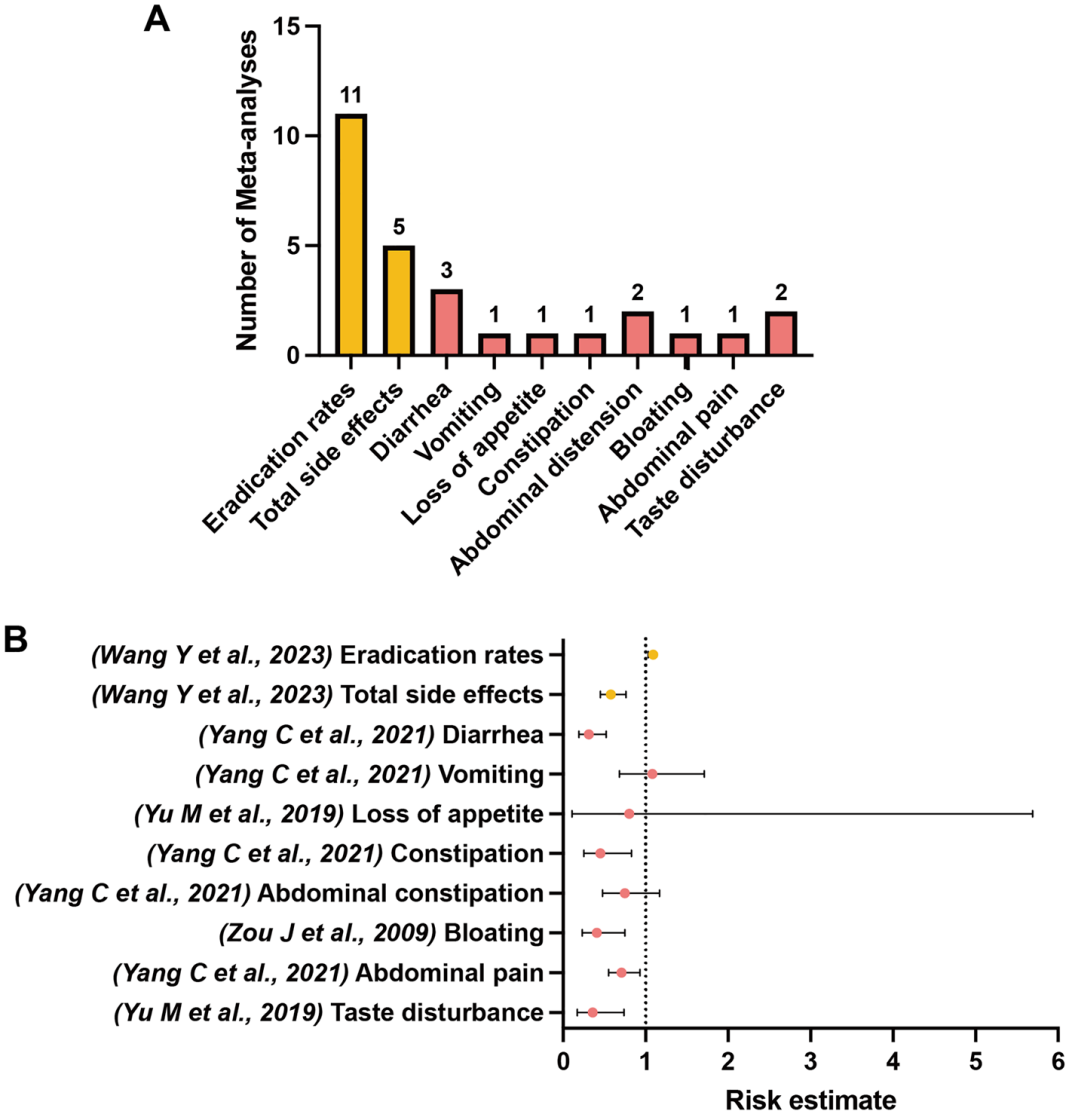
(**A**) Number of systematic reviews with meta-analyses included which investigated outcomes of interest between *H. pylori* standard treatment supplemented with *Lactobacillus* spp. and the control. (**B**) Simplified forest plot summarizing evidence for the association of *H. pylori* standard treatment supplemented with *Lactobacillus* spp. with outcomes of interest in systematic reviews with meta-analyses categorized as the most comprehensive for each outcome.

**Figure 4:**
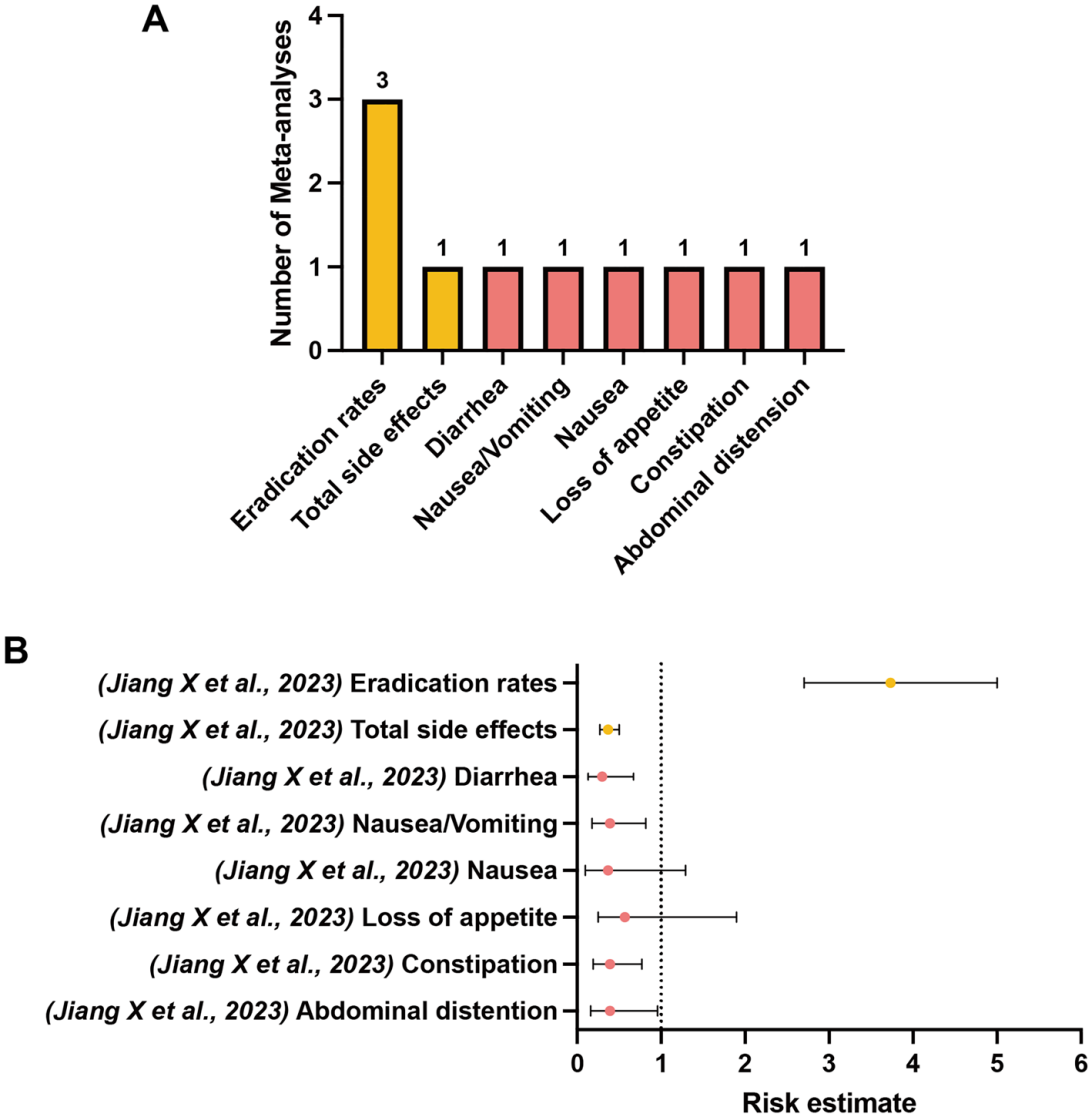
(**A**) Number of systematic reviews with meta-analyses included which investigated outcomes of interest between *H. pylori* standard treatment supplemented with *Bifidobacterium* spp. and the control. (**B**) Simplified forest plot summarizing evidence for the association of *H. pylori* standard treatment supplemented with *Bifidobacterium* spp. with outcomes of interest in systematic reviews with meta-analyses categorized as the most comprehensive for each outcome.

**Figure 5:**
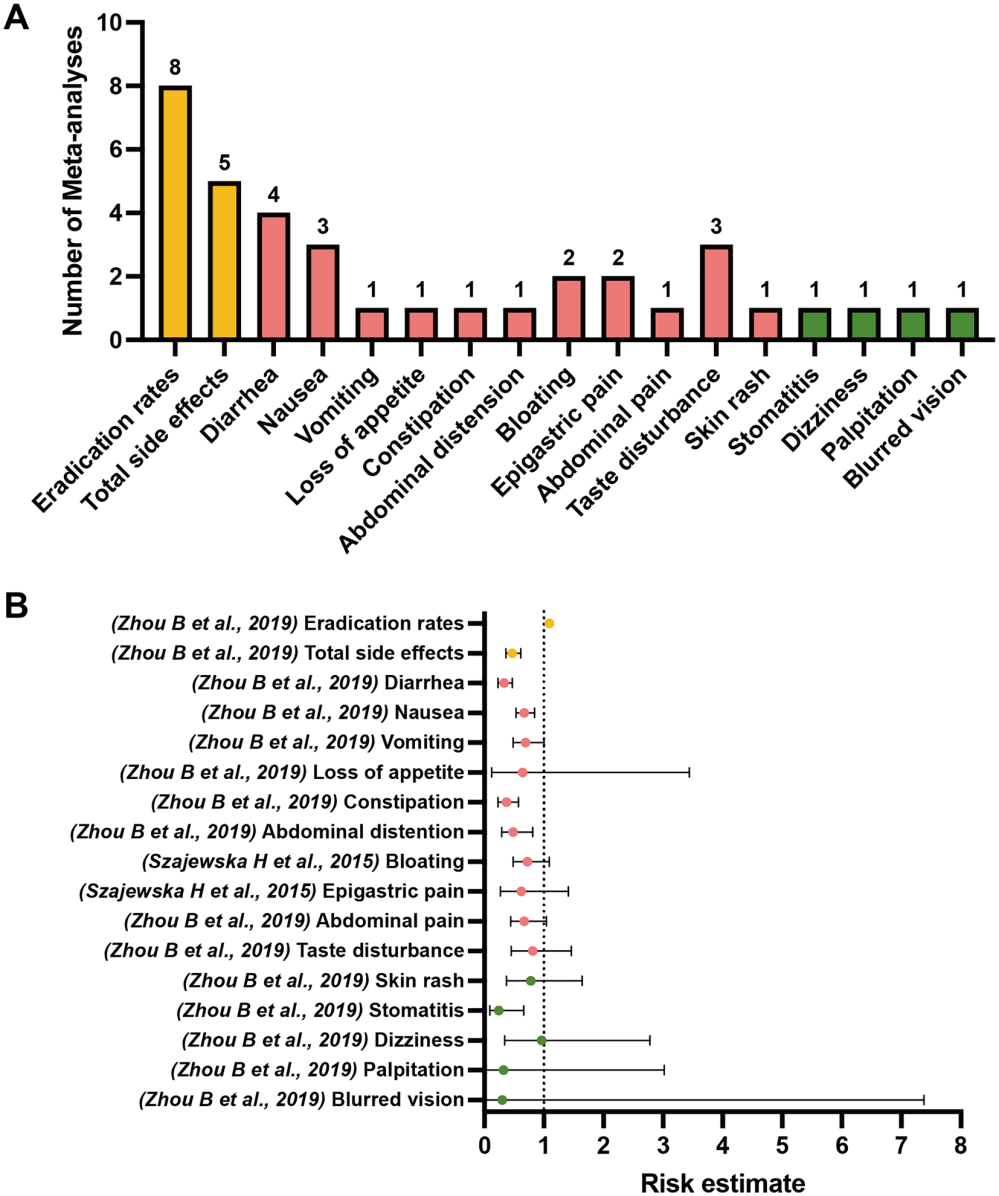
(**A**) Number of systematic reviews with meta-analyses included which investigated outcomes of interest between *H. pylori* standard treatment supplemented with *Saccharomyces* spp. and the control. (**B**) Simplified forest plot summarizing evidence for the association of *H. pylori* standard treatment supplemented with *Saccharomyces* spp. with outcomes of interest in systematic reviews with meta-analyses categorized as the most comprehensive for each outcome.

**Figure 6:**
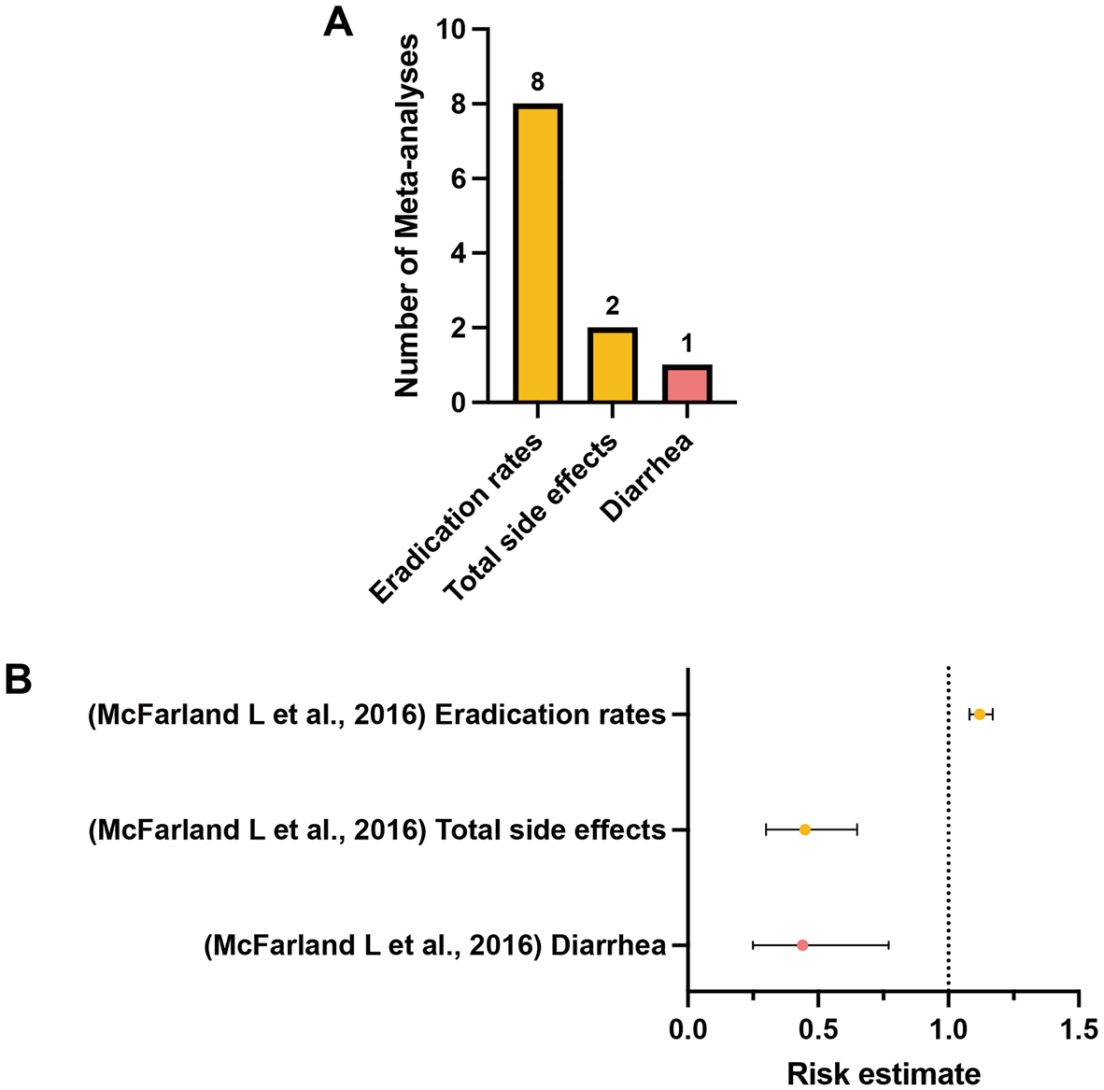
(**A**) Number of systematic reviews with meta-analyses included which investigated outcomes of interest between *H. pylori* standard treatment supplemented with multi-strained preparation and the control. (**B**) Simplified forest plot summarizing evidence for the association of *H. pylori* standard treatment supplemented with multi-strained preparation with outcomes of interest in systematic reviews with meta-analyses categorized as the most comprehensive for each outcome.

Among the included meta-analyses, 20 unique outcomes of interest classified into the following four categories were extracted: eradication rates (marked in orange), total side effects (marked in orange), specific GI side effects (marked in pink), and other nonspecific side effects (marked in green), as shown in Figs. 2, 3, 4, 5 and 6. We scrutinized every single outcome of interest from single-strained preparation (*Lactobacillus* spp., *Bifidobacterium* spp., *Saccharomyces* spp.), combined preparation (multi-strained), and the pooled probiotic strains. Most of the included meta-analyses reported on eradication rates and total side effects, but fewer studies have concurrently analyzed these outcomes for either single-strained or multi-strained preparations. Besides, sample sizes varied widely across the included articles, from dozens to thousands. Table S2 summaries details of the included studies. After quality assessment of evidence through AMSTAR 2, only one study was assessed “moderate” quality^35^, and three studies were assessed “low” quality^38,39,44^, with the remaining classified as “critically low” quality. Table S3 delineates the comprehensive details of the AMSTAR 2 assessment for each meta-analysis included in our study. Our findings indicate a prevalent issue with the declaration of methods prior to the conduct of the review. Out of 28 meta-analyses assessed, a significant majority (24 meta-analyses) failed to affirmatively state their methods before the review launched. Only 4 meta-analyses complied with this criterion^35,38,39,44^. This lack of upfront methodological transparency raises concerns about the potential for post hoc decision-making, which could introduce bias and undermine the reliability of the meta-analysis findings. Another area of concern pertains to the listing of excluded reviews. Our assessment revealed that 26 out of 28 meta-analyses did not provide a satisfactory explanation for the literature they excluded from their analysis. Conversely, only 2 meta-analyses managed to meet this standard by adequately justifying their selection and exclusion criteria^24,35^. This discrepancy underscores the need for more rigorous documentation and transparency in the review process, ensuring that readers and future researchers can fully understand the scope of the literature considered and the rationale behind exclusions.

### Outcome of interest in pooled probiotic strains

A meta-analysis including 25 primary RCT studies conducted by McFarland et al.^35^ was selected as the most comprehensive study bearing the highest AMSTAR 2 rating among all the other meta-analyses. The research team found that probiotics supplementation with pooled probiotic strains was significantly associated with increased eradication rates versus the control group (RR 1.10, 95% CI 1.06–1.14) (moderate; IV)^35^. At the first mention of this result, it is evaluated under the AMSTAR 2 assessment tool^22^, which includes 16 items and generates an overall quality rating from “high,” “moderate,” “low,” to “critically low” based on deficiencies in critical domains. The quality of this study was determined to be “moderate.” Furthermore, the evidence of outcomes was categorized into one of five levels according to evidence classification criteria^23^, with this study’s evidence being classified as Class IV, indicating “weak evidence.” This means that, although an association is observed, more research is needed to establish this conclusively.

The comparison of the probiotic supplementation with pooled probiotic strains and the control indicated a notable decreased risk of total side effects (RR 0.54, 95% CI 0.42–0.70) (moderate; IV)^35^. Specifically, probiotics supplementation were found significantly associated with lower risk of diarrhea (low; IV)^35^, nausea/vomiting (low; IV)^38^, nausea (critically low; III)^14^, vomiting (critically low; IV)^14^, constipation (low; IV)^38^, abdominal pain (critically low; IV)^13^, and taste disturbance (critically low; IV)^14^ compared to the control group. However, probiotics supplementation with pooled probiotic strains was not linked to lower risk of loss of appetite^38^, abdominal distension^38^, bloating^13^, epigastric pain^38^, metallic taste^13^, flatus^13^, skin rash^13^, headache^13^, and dizziness^13^, as compared to the control group (Fig. 2) (Table S4).

### Outcome of interest in single-strained preparation

Despite all meta-analyses on *Lactobacillus*-supplemented probiotics for *H. pylori* treatment receiving critically low AMSTAR 2 ratings, the analysis conducted by Wang et al.^47^ stood out as the most comprehensive study. This selection was based on its recency and the inclusion of the highest number of RCTs. The *Lactobacillus*-supplemented standard eradication therapy versus control was found associated with slightly higher eradication rates (79.8% vs. 72.8%, RR 1.09, 95% CI 1.03–1.15) (critically low; IV)^47^ with significant difference, and with lower risk of total side effects (20.7% vs. 40.3%, RR 0.58, 95% CI 0.45–0.76) (critically low; IV)^47^. In terms of specific GI side effects, the most comprehensive meta-analysis showed that supplementation of *Lactobacillus* spp. in *H. pylori* standard therapy was conducive to lower the risk of diarrhea by 69% (RR 0.31, 95% CI 0.19–0.52) (critically low; IV)^45^. The risk estimate of constipation^45^, bloating^26^, abdominal pain^13^, and taste disturbance^14^ in *Lactobacillus*-supplemented group reported by several meta-analyses (critically low; IV) showed varying degrees of decline, as compared to the control group (Fig. 3) (Table S4).

The meta-analysis conducted by Jiang et al.^46^ was selected as the most comprehensive study for single-strained preparation of *Bifidobacterium* spp. due to its recency and the inclusion of the highest number of RCTs. Notably, among different single-strained preparations, supplementation of *Bifidobacterium* spp. in *H. pylori* standard treatment exhibited great potential to eradicate pathogens, demonstrating a significant higher odds of curative outcome (OR 3.73, 95% CI 2.79–5.00) (critically low; III)^46^ as compared to the control. A significant lower odds of total side effects (OR 0.37, 95% CI 0.27–0.50) (critically low; III)^46^ was also found. Specifically, supplementation of *Bifidobacterium* spp. was significantly associated with lower odds of diarrhea (critically low; IV)^46^, nausea/vomiting (critically low; IV)^46^, constipation (critically low; IV)^46^, and abdominal distension (critically low; IV)^46^, while the odds of nausea (critically low; NS)^46^ and loss of appetite (critically low; NS)^46^ did not significantly decline between the experimental group and the control group (Fig. 4) (Table S4). Collectively, there was no other nonspecific side effect reported in *Lactobacillus*-supplemented or *Bifidobacterium*-supplemented standard therapy versus control.

Despite two meta-analyses presented by Lv et al.^34^ and Wang et al.^47^ suggesting that *Saccharomyces* spp. supplementation during *H. pylori* treatment was not associated with enhanced eradication rates, the most comprehensive meta-analysis conducted by Zhou et al.^44^ contradicted the hypothesis. The meta-analysis, despite lacking the most recent data, but possessing the highest AMSTAR 2 rating and the greatest number of RCTs, revealed that the eradication rates were significantly higher in the *Saccharomyces spp.* supplementation group versus the control group (81.8% vs. 74.3%, RR 1.09, 95% CI 1.05–1.13) (low; III)^44^. Besides, Zhou et al.^44^ reported lower risk of total side effects (RR 0.47, 95% CI 0.36–0.61) (low; III) using standard treatment supplemented with *Saccharomyces* spp. Although the risk of most side effects occurred during *Saccharomyces*-supplemented *H. pylori* eradication was not significantly reduced^27,36,44^, the risk of specific GI side effects such as diarrhea (critically low; III)^44^, nausea (low; III)^44^, constipation (low; IV)^44^, abdominal distension (low; IV)^44^, and stomatitis (low; IV)^44^ as a nonspecific side effect was decreased in varying degrees, as compared to the control (Fig. 5) (Table S4).

### Outcome of interest in multi-strained preparation

Risk estimates regarding the effects of multi-strained probiotics supplementation on *H. pylori* eradication rates were available for eight meta-analyses. The meta-analyses conducted by McFarland et al.^39^ bearing the highest AMSTAR 2 rating was selected as the most comprehensive study. The results showed that eradication rates improved by 12% in the experimental group (85.8%) compared with the control group (76.8%) and that the RR was 1.12 (95% CI 1.08–1.17) (low; IV)^39^. Also, the risk of total side effects decreased by 55% in the multi-strained probiotics supplementation group compared with the control group. The RR for the experimental group was 0.45 (95% CI 0.30–0.65) (low; IV)^39^. In terms of specific side effects, they noted a significant decrease in the risk of diarrhea (RR 0.44, 95% CI 0.25–0.77) (low, IV)^39^ in the multi-strained probiotics supplementation group compared with the control group. No other nonspecific side effects were reported in association with the multi-strained preparation during the standard treatment of *H. pylori* as compared to the control (Fig. 6) (Table S4).

## Discussion

Our comprehensive umbrella review collates evidence from 28 unique meta-analyses based on 534 RCTs, highlighting an association between probiotics supplementation and improved eradication rates and reduced total side effects during *H. pylori* standard treatment. These findings, in alignment with previous systematic reviews with meta-analyses, further solidify the growing body of evidence indicating the beneficial use of probiotics in enhancing the eradication rates of *H. pylori* and reducing the incidence of related side effects.

The evidence demonstrates a significant improvement in *H. pylori* eradication rates with probiotics supplementation when used alongside standard therapy. It aligns with the conclusions of another umbrella review, which exclusively examined the effect of probiotics supplementation on *H. pylori* eradication without considering its impact on reducing side effects^49^. This improvement is especially crucial considering the prevailing issues with the standard triple or quadruple regimens^12–14^, which confront with reduced effectiveness due to rising antibiotic resistance^5,6^. Probiotics supplementation, by providing a means of modulating the gastrointestinal microbiota, may offer a new approach to combatting such resistance^9^. Furthermore, their capacity to alleviate common antibiotic-associated side effects, such as diarrhea, nausea, and vomiting, may improve patient adherence to therapy^7^.

The observed variation in outcomes between different probiotic genera suggests that specific genera or combinations thereof may be more effective in treating *H. pylori*. Notably, supplementation with *Bifidobacterium* spp. showed greater effectiveness as a single-strained preparation at the genus level for *H. pylori* eradication, while *Lactobacillus* spp. and *Saccharomyces* spp. also exhibited beneficial effects. This underscores the possibility of tailoring probiotic supplementation based on genus-specific effects, which could optimize *H. pylori* eradication rates and minimize side effects. It is imperative to acknowledge that the efficacy of probiotics is highly strain-specific, and not all strains within a genus may offer the same health benefits^16^. This strain-specificity is critical for understanding the mechanisms of action and for guiding the selection of probiotic strains in clinical practice. Future research should further investigate the mechanisms underlying these genus-specific effects and the potential synergistic effects of multi-strained probiotics.

The utilization of probiotics could be considered as supplementary therapy for *H. pylori* eradication primarily due to three distinct capabilities: (1) they foster mucin production, which, in turn, restricts the pathogen's adhesion to the gut surface; (2) they generate short-chain fatty acids and other antimicrobial substances, potentially decreasing the density of *H. pylori*; and (3) they offer protection against human pathogens through host receptor competition and immune modulation mechanisms^50^. Recent decades have witnessed an abundance of in vitro studies demonstrating that *Bifidobacterium* spp. could inhibit pathogens using a variety of mechanisms such as production of organic acids, antibacterial peptides, and quorum-sensing inhibitors, as well as immune stimulation^51–54^. These methods collectively offer molecular evidence highlighting their inherent potential to prevent *H. pylori* infections. For instance, certain strains of *Bifidobacterium* spp. are known to produce acetate, which has been demonstrated to play a key protective role against some infectious diseases^52^. Adding to this, a recent study found that zinc acetate can increase the sensitivity of *H. pylori* to levofloxacin, an antibiotic commonly used in *H. pylori* eradication regimens^55^. This provides a possible mechanism for enhancing the treatment of *H. pylori* by increasing the acetate produced by *Bifidobacterium* spp., which probably could explain why supplementation of *Bifidobacterium* spp. might show greater capacity for pathogen eradication compared to other probiotic strains. However, these potential mechanisms, based on in vitro studies and animal models, require further confirmation in human clinical trials.

Although the results of this umbrella review are promising, they should be interpreted with caution due to several limitations. The quality of the included systematic reviews with meta-analyses was critically low for most studies, as determined by the AMSTAR 2 criteria. Also, the considerable heterogeneity among the included meta-analyses may have influenced the outcomes. Plus, there are still unanswered questions regarding the optimal genus/strains, dosages, and duration of probiotics for *H. pylori* treatment, and the specific mechanisms through which they impact the potential probiotics-gut microbiota cross talk and immune system remain unclear. Moreover, further exploration in high-quality design is necessary to determine whether certain subgroups of patients might benefit more from probiotic supplementation, taking into account factors such as age, disease severity, recurrence rate and antibiotic resistance profiles.

To enhance the quality of systematic reviews with meta-analyses in future research, it is essential to address these limitations. First, adopting rigorous methodologies that adhere closely to established guidelines, such as those outlined in the AMSTAR 2, can significantly improve study quality. As shown in Table S3, most of the meta-analyses failed to meet the requirements in question 2 (24/28), 7 (26/28), and 10 (26/28), which ultimately leads to poor AMSTAR 2 rating. Efforts should be made to minimize heterogeneity among studies by clearly defining inclusion criteria, intervention protocols, and outcome measures. Additionally, there is a pressing need for more comprehensive data on probiotics’ specific strains, dosages, and treatment durations, necessitating targeted research in these areas. Furthermore, future reviews should place a greater emphasis on subgroup analyses to identify patient populations that may derive the most benefit from probiotic supplementation. This involves a more detailed examination of factors such as age, disease severity, recurrence rates, and antibiotic resistance profiles. Incorporating such analyses can help in tailoring probiotic supplementation strategies to individual patient needs, potentially leading to more effective management of *H. pylori* infection.

In conclusion, probiotics supplementation is a plausible adjunctive strategy in the management of *H. pylori*, with potential benefits in terms of eradication rates improvement and side effects reduction. Until rigorous evidence is available, clinicians should consider probiotics as a promising, albeit not definitively proven, adjunct to *H. pylori* eradication therapy. Besides, our umbrella review underscores the need for well-designed, high-quality RCTs and systematic reviews with meta-analyses that will further validate the effectiveness of probiotics, identify optimal genus/strains and combinations, and determine their best therapeutic application within the clinical setting. This may also pave the way for personalized probiotics supplementation strategies in the management of *H. pylori* infection in future.

### Supplementary Information

Supplementary Table S1.

Supplementary Table S2.

Supplementary Table S3.

Supplementary Table S4.

## Data Availability

The datasets generated or analyzed in the current study are available from the corresponding author upon reasonable request.
