# Supplementary material for: The effects of probiotics supplementation on Helicobacter pylori standard treatment: an umbrella review of systematic reviews with meta-analyses
Source: Sci Rep. 2024 May 2;14:10069. doi: 10.1038/s41598-024-59399-4 (PMC11066092; doi:10.1038/s41598-024-59399-4)
Supplement: Supplementary file 1 — Supplementary Table S1. [file 41598_2024_59399_MOESM1_ESM.docx]

**Table S1** Search strategy.

| **Databases** | **Search items** |
| --- | --- |
| PubMed | #1: “helicobacter pylori”[MeSH Terms] OR “helicobacter infections”[MeSH Terms]  #2: “helicobacter pylori”[Title/Abstract] OR “helicobacter infection*”[Title/Abstract] OR “Hp”[Title/Abstract] OR “H. pylori”[Title/Abstract] OR “h. pylori”[Title/Abstract]  #3: #1 OR #2  #4: “probiotics”[MeSH Terms] OR “synbiotics”[MeSH Terms]  #5: “probiotic*”[Title/Abstract] OR “synbiotic*”[Title/Abstract] OR “Lactobacillus”[Title/Abstract] OR “Lactococcus”[Title/Abstract] OR “Bacillus”[Title/Abstract] OR “Clostridium”[Title/Abstract] OR “Saccharomyces”[Title/Abstract] OR “Streptococcus”[Title/Abstract] OR “Bifidobacterium”[Title/Abstract] OR “Enterococcus”[Title/Abstract] OR “Bioflor”[Title/Abstract]  #6: #4 OR #5  #7: #3 AND #6  #8: “meta-analysis”[Publication Type] OR “systematic review”[Publication Type]  #9: #7 AND #8 |
| Embase | #1: ‘helicobacter pylori’/exp OR ‘helicobacter infection’/exp  #2: ‘helicobacter pylori’:ab,ti OR ‘helicobacter infection*’:ab,ti OR ‘Hp’:ab,ti OR ‘H. pylori’:ab,ti OR ‘h. pylori’:ab,ti  #3: #1 OR #2  #4: ‘probiotic agent’/exp OR ‘synbiotic agent’/exp  #5: ‘probiotic*’:ab,ti OR ‘synbiotic*’:ab,ti OR ‘Lactobacillus’:ab,ti OR ‘Lactococcus’:ab,ti OR ‘Bacillus’:ab,ti OR ‘Clostridium’:ab,ti OR ‘Saccharomyces’:ab,ti OR ‘Streptococcus’:ab,ti OR ‘Bifidobacterium’:ab,ti OR ‘Enterococcus’:ab,ti OR ‘Bioflor’:ab,ti  #6: #4 OR #5  #7: #3 AND #6  #8: ‘meta-analysis’:ab,ti OR ‘systematic review’:ab,ti  #9: #7 AND #8 |
| Cochrane  Library | #1: MeSH descriptor: [Helicobacter pylori] explode all trees  #2: MeSH descriptor: [Helicobacter infections] explode all trees  #3: helicobacter pylori:ti,ab,kw OR helicobacter infection*:ti,ab,kw OR Hp:ti,ab,kw OR H. pylori:ti,ab,kw OR h. pylori:ti,ab,kw  #4: #1 OR #2 OR #3  #5: MeSH descriptor: [Probiotics] explode all trees  #6: MeSH descriptor: [Synbiotics] explode all trees  #7: probiotic*:ti,ab,kw OR synbiotic*:ti,ab,kw OR Lactobacillus:ti,ab,kw OR Lactococcus:ti,ab,kw OR Bacillus:ti,ab,kw OR Clostridium:ti,ab,kw OR Saccharomyces:ti,ab,kw OR Streptococcus:ti,ab,kw OR Bifidobacterium:ti,ab,kw OR Enterococcus:ti,ab,kw OR Bioflor:ti,ab,kw  #8: #5 OR #6 OR #7  #9: #4 AND #8  #10: meta-analysis:ti,ab,kw OR systematic review:ti,ab,kw  #11: #9 AND #10 |
