# Supplementary material for: The effects of probiotics supplementation on Helicobacter pylori standard treatment: an umbrella review of systematic reviews with meta-analyses
Source: Sci Rep. 2024 May 2;14:10069. doi: 10.1038/s41598-024-59399-4 (PMC11066092; doi:10.1038/s41598-024-59399-4)
Supplement: Supplementary file 2 — Supplementary Table S2. [file 41598_2024_59399_MOESM2_ESM.docx]

**Table S2** Description of systematic reviews with meta-analyses included in the umbrella review.

| **Study** | **Outcome investigated** | **Subgroup investigated** | **RCT studies regarding eradication rates (n)** | **Selection as the most comprehensive** | **Overall AMSTAR 2 Rating** |
| --- | --- | --- | --- | --- | --- |
|  |  |  |  |  |  |
|  |  |  |  |  |  |
| Tong J et al., 2007^1^ | Eradication rates Total side effects Diarrhea Epigastric pain Nausea Taste disturbance | *Lactobacillus spp. Bacillus spp. Clostridium spp.* Combined preparation | 11 | - | Critically low |
| Sachdeva A et al., 2009^2^ | Eradication rates Total side effects | - | 10 | - | Critically low |
| Wang Z et al., 2013^3^ | Eradication rates Total side effects | - | 10 | - | Critically low |
| Zhu R et al., 2014^4^ | Eradication rates Total side effects Diarrhea Taste disturbance Metallic taste Vomiting Nausea Epigastric pain | - | 14 | - | Critically low |
| Dang Y et al., 2014^5^ | Eradication rates Total side effects | - | 33 | - | Critically low |
| Li B et al., 2015^6^ | Eradication rates Total side effects | - | 11 | - | Critically low |
| Lv Z et al., 2015^7^ | Eradication rates Total side effects Diarrhea Nausea/Vomiting Epigastric pain | *Lactobacillus spp. Saccharomyces spp. Bifidobacterium spp.* Combined preparation | 21 | - | Critically low |
| Gong Y et al., 2015^8^ | Eradication rates  Nausea  Diarrhea  Epigastric pain  Vomiting  Taste disturbance  Loss of appetite  Bloating  Constipation  Skin rash | *Lactobacillus spp.*  *Saccharomyces spp.*  Combined preparation | 23 | - | Critically low |
| McFarland L et al., 2015^9^ | Eradication rates Total side effects  Diarrhea | *Saccharomyces spp.*  *Clostridium spp.*  *Lactobacillus spp.* | 25 | [Pooled strains] Eradication rates  Total side effects  Diarrhea | Moderate |
| Zhang M et al., 2015^10^ | Eradication rates Total side effects | - | 45 | - | Critically low |
| Zhou B et al., 2016^11^ | Eradication rates Total side effects Diarrhea Abdominal pain Nausea/Vomiting Bloating Constipation Taste disturbance | - | 11 | - | Critically low |
| Lü M et al., 2016^12^ | Eradication rates Total side effects Nausea/Vomiting Diarrhea Constipation Epigastric pain Loss of appetite Abdominal distention | *Lactobacillus spp.* Combined preparation | 13 | [Pooled strains]  Nausea/Vomiting  Loss of appetite  Constipation  Abdominal distension  Epigastric pain | Low quality |
| Lu C et al., 2016^13^ | Eradication rates | - | 19 | - | Critically low |
| Lau C et al., 2016^14^ | Eradication rates  Nausea  Vomiting  Diarrhea  Epigastric pain | *Lactobacillus spp.*  *Saccharomyces spp.*  *Bifidobacterium spp.*  Combined preparation | 31 | - | Critically low |
| Si X et al., 2017^15^ | Eradication rates Total side effects | *Lactobacillus spp.* Combined preparation | 16 | - | Critically low |
| Wang F et al., 2017^16^ | Eradication rates Total side effects | - | 140 | - | Critically low |
| Shi X et al., 2019^17^ | Eradication rates Total side effects Diarrhea Abdominal pain Nausea Taste disturbance Vomiting Constipation | - | 40 | [Pooled strains] Nausea Vomiting Taste disturbance | Critically low |
| Zhang M et al., 2020^18^ | Eradication rates  Total side effects  Nausea/Vomiting  Diarrhea  Constipation  Epigastric pain  Taste disturbance  Skin rash  Loss of appetite  Bloating  Abdominal pain  Headache  Abdominal distension  Flatus  Metallic taste  Dizziness | Combined preparation | 47 | [Pooled strains]  Bloating  Abdominal pain  Metallic taste  Flatus  Skin rash  Headache  Dizziness  [Combined preparation]  Eradication rates  Total side effects | Critically low |
| Wang Y et al., 2023^19^ | Eradication rates Total side effects | *Lactobacillus spp. Bacillus spp. Saccharomyces spp.* Combined preparation | 40 | [*Lactobacillus spp.*] Eradication rates Total side effects | Critically low |
| Zou J et al., 2009^20^ | [*Lactobacillus spp.*] Eradication rates Total side effects Diarrhea Bloating Taste disturbance | - | 8 | [*Lactobacillus spp.*] Bloating | Critically low |
| Zheng X et al., 2013^21^ | [*Lactobacillus spp.*]  Eradication rates  Total side effects | - | 9 | - | Critically low |
| Yu M et al., 2019^22^ | [*Lactobacillus spp.*] Eradication rates Total side effects Diarrhea Taste disturbance Abdominal distension Loss of appetite | - | 11 | [*Lactobacillus spp.*] Loss of appetite Taste disturbance | Critically low |
| Yang C et al., 2021^23^ | [*Lactobacillus spp.*] Eradication rates Total side effects Diarrhea Abdominal pain Vomiting Constipation Abdominal distension | - | 5 | [*Lactobacillus spp.*] Diarrhea Vomiting Constipation Abdominal distension Abdominal pain | Critically low |
| Jiang X et al., 2023^24^ | [*Bifidobacterium spp.*] Eradication rates Total side effects Loss of appetite Abdominal distension Diarrhea Nausea/Vomiting Nausea Constipation | - | 16 | [*Bifidobacterium spp.*] Eradication rates Total side effects  Diarrhea Loss of appetite Abdominal distension Nausea/Vomiting Nausea Constipation | Critically low |
| Szajewska H et al., 2010^25^ | [*Saccharomyces spp.*]  Eradication rates  Total side effects  Diarrhea  Epigastric pain  Taste disturbance  Nausea  Bloating | - | 4 | - | Critically low |
| Szajewska H et al., 2015^26^ | [*Saccharomyces spp.*] Eradication rates Total side effects Diarrhea Epigastric pain Taste disturbance Nausea Bloating | - | 9 | [*Saccharomyces spp.*] Bloating Epigastric pain | Critically low |
| Zhou B et al., 2019^27^ | [*Saccharomyces spp.*]  Eradication rates  Total side effects  Diarrhea  Nausea  Vomiting  Constipation  Abdominal distention  Abdominal pain  Taste disturbance  Loss of appetite  Stomatitis  Skin rash  Dizziness  Palpitation  Blurred vision | - | 17 | [*Saccharomyces spp.*]  Eradication rates  Total side effects  Diarrhea  Nausea  Vomiting  Constipation  Abdominal distention  Abdominal pain  Taste disturbance  Loss of appetite  Stomatitis  Skin rash  Dizziness  Palpitation  Blurred vision | Low quality |
| McFarland L et al., 2016^28^ | [Combined preparation] Eradication rates Total side effects Diarrhea | - | 20 | [Combined preparation] Eradication rates Total side effects Diarrhea | Low quality |

1. Tong JL, Ran ZH, Shen J, Zhang CX, Xiao SD. Meta-analysis: the effect of supplementation with probiotics on eradication rates and adverse events during Helicobacter pylori eradication therapy. *Aliment Pharmacol Ther*. Jan 15 2007;25(2):155-68. doi:10.1111/j.1365-2036.2006.03179.x

2. Sachdeva A, Nagpal J. Effect of fermented milk-based probiotic preparations on Helicobacter pylori eradication: a systematic review and meta-analysis of randomized-controlled trials. *Eur J Gastroenterol Hepatol*. Jan 2009;21(1):45-53. doi:10.1097/MEG.0b013e32830d0eff

3. Wang ZH, Gao QY, Fang JY. Meta-analysis of the efficacy and safety of Lactobacillus-containing and Bifidobacterium-containing probiotic compound preparation in Helicobacter pylori eradication therapy. *J Clin Gastroenterol*. Jan 2013;47(1):25-32. doi:10.1097/MCG.0b013e318266f6cf

4. Zhu R, Chen K, Zheng YY, et al. Meta-analysis of the efficacy of probiotics in Helicobacter pylori eradication therapy. *World J Gastroenterol*. Dec 21 2014;20(47):18013-21. doi:10.3748/wjg.v20.i47.18013

5. Dang Y, Reinhardt JD, Zhou X, Zhang G. The effect of probiotics supplementation on Helicobacter pylori eradication rates and side effects during eradication therapy: a meta-analysis. *PLoS One*. 2014;9(11):e111030. doi:10.1371/journal.pone.0111030

6. Li BZ, Threapleton DE, Wang JY, et al. Comparative effectiveness and tolerance of treatments for Helicobacter pylori: systematic review and network meta-analysis. *BMJ*. Aug 19 2015;351:h4052. doi:10.1136/bmj.h4052

7. Lv Z, Wang B, Zhou X, et al. Efficacy and safety of probiotics as adjuvant agents for Helicobacter pylori infection: A meta-analysis. *Exp Ther Med*. Mar 2015;9(3):707-716. doi:10.3892/etm.2015.2174

8. Gong Y, Li Y, Sun Q. Probiotics improve efficacy and tolerability of triple therapy to eradicate Helicobacter pylori: a meta-analysis of randomized controlled trials. *Int J Clin Exp Med*. 2015;8(4):6530-43.

9. McFarland LV, Malfertheiner P, Huang Y, Wang L. Meta-analysis of single strain probiotics for the eradication of Helicobacter pylori and prevention of adverse events. *World Journal of Meta-analysis*. 2015;3(2):97-117.

10. Zhang MM, Qian W, Qin YY, He J, Zhou YH. Probiotics in Helicobacter pylori eradication therapy: a systematic review and meta-analysis. *World J Gastroenterol*. Apr 14 2015;21(14):4345-57. doi:10.3748/wjg.v21.i14.4345

11. Zhou BG, Cheng QJ, Liu M, Guo P, Xiao Z, Chen AH. Probiotics-containing Rescue Regimen for the Eradication of Helicobacter Pylori Infection: A Systematic Review. *Chin J Evid-based Med*. 2016;16(05):550-556.

12. Lu M, Yu S, Deng J, et al. Efficacy of Probiotic Supplementation Therapy for Helicobacter pylori Eradication: A Meta-Analysis of Randomized Controlled Trials. *PLoS One*. 2016;11(10):e0163743. doi:10.1371/journal.pone.0163743

13. Lu C, Sang J, He H, et al. Probiotic supplementation does not improve eradication rate of Helicobacter pylori infection compared to placebo based on standard therapy: a meta-analysis. *Sci Rep*. Mar 21 2016;6:23522. doi:10.1038/srep23522

14. Lau CS, Ward A, Chamberlain RS. Probiotics improve the efficacy of standard triple therapy in the eradication of Helicobacter pylori: a meta-analysis. *Infect Drug Resist*. 2016;9:275-289. doi:10.2147/IDR.S117886

15. Si X, Lan Y, Qiao L. A meta-analysis of randomized controlled trials of bismuth-containing quadruple therapy combined with probiotic supplement for eradication of Helicobacter pylori. *Chin J Intern Med*. 2017;56(10):752-759. doi:10.3760/cma.j.issn.0578-1426.2017.10.009

16. Wang F, Feng J, Chen P, et al. Probiotics in Helicobacter pylori eradication therapy: Systematic review and network meta-analysis. *Clin Res Hepatol Gastroenterol*. Sep 2017;41(4):466-475. doi:10.1016/j.clinre.2017.04.004

17. Shi X, Zhang J, Mo L, Shi J, Qin M, Huang X. Efficacy and safety of probiotics in eradicating Helicobacter pylori: A network meta-analysis. *Medicine (Baltimore)*. Apr 2019;98(15):e15180. doi:10.1097/MD.0000000000015180

18. Zhang M, Zhang C, Zhao J, Zhang H, Zhai Q, Chen W. Meta-analysis of the efficacy of probiotic-supplemented therapy on the eradication of H. pylori and incidence of therapy-associated side effects. *Microb Pathog*. Oct 2020;147:104403. doi:10.1016/j.micpath.2020.104403

19. Wang Y, Wang X, Cao XY, Zhu HL, Miao L. Comparative effectiveness of different probiotics supplements for triple helicobacter pylori eradication: a network meta-analysis. *Front Cell Infect Microbiol*. 2023;13:1120789. doi:10.3389/fcimb.2023.1120789

20. Zou J, Dong J, Yu X. Meta-analysis: Lactobacillus containing quadruple therapy versus standard triple first-line therapy for Helicobacter pylori eradication. *Helicobacter*. Oct 2009;14(5):97-107. doi:10.1111/j.1523-5378.2009.00716.x

21. Zheng X, Lyu L, Mei Z. Lactobacillus-containing probiotic supplementation increases Helicobacter pylori eradication rate: evidence from a meta-analysis. *Rev Esp Enferm Dig*. Sep 2013;105(8):445-53. doi:10.4321/s1130-01082013000800002

22. Yu M, Zhang R, Ni P, Chen S, Duan G. Efficacy of Lactobacillus-supplemented triple therapy for H. pylori eradication: A meta-analysis of randomized controlled trials. *PLoS One*. 2019;14(10):e0223309. doi:10.1371/journal.pone.0223309

23. Yang C, Liu L, Majaw JK, Liang L, Chen Y. Efficacy of Lactobacillus reuteri supplementation therapy for Helicobacter pylori eradication: A meta-analysis of randomised controlled trials. *Medicine in Microecology*. 2021/06/01/ 2021;8:100036. doi:<https://doi.org/10.1016/j.medmic.2021.100036>

24. Jiang X, Xu C, Liu B, Chen P, Xu Q, Zhang L. Efficacy and safety of bifidobacterium quadruple viable tablets in the treatment of Helicobacter pylori-infected peptic ulcer or gastritis patients: a systematic review and meta-analysis. *BMC Infect Dis*. May 9 2023;23(1):313. doi:10.1186/s12879-023-08211-1

25. Szajewska H, Horvath A, Piwowarczyk A. Meta-analysis: the effects of Saccharomyces boulardii supplementation on Helicobacter pylori eradication rates and side effects during treatment. *Aliment Pharmacol Ther*. Nov 2010;32(9):1069-79. doi:10.1111/j.1365-2036.2010.04457.x

26. Szajewska H, Horvath A, Kolodziej M. Systematic review with meta-analysis: Saccharomyces boulardii supplementation and eradication of Helicobacter pylori infection. *Aliment Pharmacol Ther*. Jun 2015;41(12):1237-45. doi:10.1111/apt.13214

27. Zhou BG, Chen LX, Li B, Wan LY, Ai YW. Saccharomyces boulardii as an adjuvant therapy for Helicobacter pylori eradication: A systematic review and meta-analysis with trial sequential analysis. *Helicobacter*. Oct 2019;24(5):e12651. doi:10.1111/hel.12651

28. McFarland LV, Huang Y, Wang L, Malfertheiner P. Systematic review and meta-analysis: Multi-strain probiotics as adjunct therapy for Helicobacter pylori eradication and prevention of adverse events. *United European Gastroenterol J*. Aug 2016;4(4):546-61. doi:10.1177/2050640615617358
